# Fat accumulation in striped hamsters (*Cricetulus barabensis*) reflects the temperature of prior cold acclimation

**DOI:** 10.1186/s12983-024-00523-5

**Published:** 2024-02-13

**Authors:** Kaiyuan Zhang, Jing Cao, Zhijun Zhao

**Affiliations:** College of Life and Environmental Science, Zhejiang Provincial Key Laboratory for Water Environment and Marine Biological Resources Protection, Wenzhou University, Wenzhou, 325035 China

**Keywords:** Body fat, Brown adipose tissue, Striped hamsters, Temperature, Metabolic thermogenesis, Uncoupling protein 1, Thyroid hormones

## Abstract

**Background:**

Proper adjustments of metabolic thermogenesis play an important role in thermoregulation in endotherm to cope with cold and/or warm ambient temperatures, however its roles in energy balance and fat accumulation remain uncertain. Our study aimed to investigate the effect of previous cold exposure (10 and 0 °C) on the energy budgets and fat accumulation in the striped hamsters (*Cricetulus barabensis*) in response to warm acclimation. The body mass, energy intake, resting metabolic rate (RMR) and nonshivering thermogenesis (NST), serum thyroid hormone levels (THs: T3 and T4), and the activity of brown adipose tissue (BAT), indicated by cytochrome c oxidase (COX) activity and uncoupling protein 1 (*ucp*_1_) expression, were measured following exposure to the cold (10 °C and 0 °C) and transition to the warm temperature (30 °C).

**Results:**

The hamsters at 10 °C and 0 °C showed significant increases in energy intake, RMR and NST, and a considerable reduction in body fat than their counterparts kept at 21 °C. After being transferred from cold to warm temperature, the hamsters consumed less food, and decreased RMR and NST, but they significantly increased body fat content. Interestingly, the hamsters that were previously exposed to the colder temperature showed significantly more fat accumulation after transition to the warm. Serum T3 levels, BAT COX activity and *ucp*_1_ mRNA expression were significantly increased following cold exposure, and were considerably decreased after transition to the warm. Furthermore, body fat content was negatively correlated with serum T3 levels, BAT COX activity and UCP_1_ expression.

**Conclusion:**

The data suggest that the positive energy balance resulting from the decreased RMR and NST in BAT under the transition from the cold to the warm plays important roles in inducing fat accumulation. The extent of fat accumulation in the warm appears to reflect the temperature of the previous cold acclimation.

## Background

Ambient temperature is known to be an important natural factor that affects the physiology and behaviour of small mammals inhabiting temperate regions [1–7]. In the field, seasonal cycles of body mass and / or body fat are important strategies in variety of rodents for coping with cold winters and hot summers [8–12]. Therefore, exposing these seasonal rodents to different temperature regimes will provide new information on the interactions between thermoregulatory energy use and body fat accumulation. Body fat adjustment is determined by a balance between energy intake and expenditure [13]. A positive energy balance results in an excessive amount of body fat in relation to lean mass, which is usually induced by an increase in energy intake, such as hyperphagia for animals when fed with high-fat diet [13, 14]. In contrast, a considerable decrease in food intake, and/ or an increase in energy expenditure may lead to the negative energy balance, and consequently the decreased fat store and lean body mass [15–18]. For example, Brandt’s voles (*Lasiopodomys brandtii*) and Mongolian gerbils (*Meriones unguiculatus*) increase energy intake in response to cold exposure but also exhibit significantly lower body mass and/or body fat [19, 20]. Djungarian hamster (*Phodopus sungorus*) exhibits seasonal changes in body mass with a maximum of about 45 g in summer and a minimum near 25 g during winter, and much of weight loss is in the form of mobilized fat reserves [12, 16, 21]. These seasonal rodents are responsive to mixed environmental factors including ambient temperature, photoperiod, food quality and availability, making it difficult to separate one factor from the others in the field. It has been widely reported that energy budget and metabolic rate of an animal are closely associated with temperature, which makes ambient temperature a potential candidate to induce the changes in body fat of these seasonal rodents, but not excluding photoperiod and food, etc. However, it’s unclear whether, or not, the negative energy balance of an animal previously exposed to cold is correlated with the fat accumulation when it becomes warm.

A considerable decrease in the energy expended for metabolic thermogenesis may play more important roles than energy intake in the regulation of body fat in warm conditions [19–22]. Metabolic thermogenesis represents the rate at which an animal oxidizes substrates to produce the energy required to grow, behave, reproduce, and survive. In mammals, resting metabolic rate (RMR) in thermoneutrality defined here as the rate of oxygen consumption of an inactive, non-reproductive, post-absorptive animal in a resting but not torpid or hibernating state [23–25]. Nonshivering thermogenesis (NST), mediated by the uncoupling protein 1 (UCP1) in the brown adipose tissue (BAT) and based on Ca^2+^-slippage by a sarcoplasmatic reticulum Ca^2+^-ATPase in skeletal muscle, is one of the major recognized forms of thermogenesis in small mammals [1, 4, 26]. Thermogenic capacity of BAT depends on the tissue’s high mitochondrial density and oxidative capacity, as well as the presence of UCP1 [14]. UCP1, a specific protein in the inner mitochondrial membrane of BAT, uncouples energy substrate oxidation from mitochondrial ATP production and hence results in the loss of potential energy as heat [14, 27]. As the terminal enzyme in oxidative phosphorylation in mitochondria, cytochrome c oxidase (COX, complex IV of the respiratory chain) is involved in mitochondrial energy metabolism [28, 29]. BAT has been proposed as a tissue primarily responsible for the resistance to body fat accumulation by burning off excess energy to maintain energy balance [30–34]. In addition, thyroid hormones (THs, 3,5,3',5'-tetraiodothyronine or thyroxine, T4; and 3,5,3'-triiodothyronine, T3) control important biological processes, including metabolism and energy balance [35, 36]. THs have central and peripheral effects on the regulation of BAT thermogenesis, within which UCP1 is essential for mediation of the effects of THs on energy balance [36–38]. It's known that THs are physiologically adjusted in relation to environmental factors, in particular ambient temperature [39]. Animals under warm temperature reduced THs secretion, resulting in decreased metabolic rate and heat production [40]. It is well known that the enhanced BAT function, such as the elevated COX activity and UCP1 gene expression, as well as THs levels, considerably increases the capacity of seasonal animals for coping with cold temperature, however, the roles of BAT physiological regulation in fat accumulation under the transition of cold to warm temperature have received less attention.

In the present study, we tested the hypothesis that a previous cold exposure is correlated with the extent of subsequent fat accumulation under warm conditions in striped hamsters (*Cricetulus barabensis*), a seasonal rodent distributed in northern China [41]. This species is subjected to marked seasonal fluctuations in ambient temperature, characterized by warm summers and cold winters [41, 42]. The food intake and metabolic thermogenesis of the hamsters increase in winter and decrease in summer, while the body fat content is significantly higher in summer compared to in winter [15]. In this study, the striped hamsters were exposed to cold temperatures (10 °C and 0 °C) for 2 weeks, which was followed by a warm exposure (30 °C) for 4 weeks. The energy intake, metabolic thermogenesis, and body fat, as well as BAT COX activity and *ucp*_1_ mRNA expression, and serum thyroid hormones levels, were measured during the cold exposure and the following warm exposure. We predicted that the decreased metabolic thermogenesis would be correlated with fat accumulation of the hamsters under the transition of the cold to the warm temperature.

## Methods and materials

### Animals

Striped hamsters used in this study were obtained from the colony maintained in the animal house at Wenzhou University. Animals were kept individually in plastic cages (29 × 15 × 18 cm^3^) with sawdust beddings. Food (standard rodent chow; produced by Beijing KeAo Feed, Beijing) and water were provided ad libitum*.* The ambient temperature was controlled at 21 ± 1 °C, and photoperiod was set at a 12: 12 h light: dark cycle (L: D, lights on at 08: 00 h). The hamster will interpret L8: D16 as short and L16: D8 as long photoperiod, but that does not make them critical. L12: D12 is equinox photoperiod. This study aimed to examine the effect of temperature transition from cold to warm on energy budget and body fat, therefore the hamsters were kept at the neutral photoperiod (L12: D12). All the procedures involving animals were reviewed and approved by the Animal Care and Use Committee of the University of Wenzhou. The methods were carried out in accordance with the approved guidelines.

### Experimental design

**Fig. 1:**
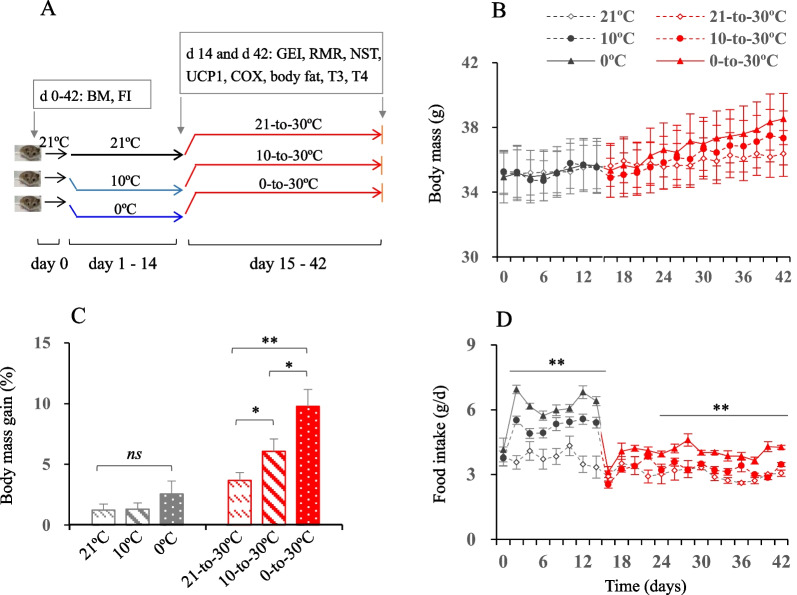
The experimental protocol, and body mass and food intake. **A** All animals were housed at an ambient temperature of 21 °C before the experiment started (day 0). The animals in 10 °C group were transferred into10°C for 14 days, and those in the 0 °C group were transferred into 0 °C for 14 days, during which the animals in 21 °C group were kept at 21 °C. At the end of day 14, half of the animals in each group were randomly selected for dissection, and the remaining animals were transferred into 30 °C from day 15 to 42 (referred to as 21–30 °C, 10–30 °C, and 0–30 °C groups). Body mass (BM) and food intake (FI) were measured on days 0–42. Gross energy intake (GEI), resting metabolic rate (RMR), non-shivering thermogenesis (NST), uncoupling protein 1 mRNA expression and cytochrome c oxidase (COX) activity of brown adipose tissue, as well as body fat content and serum T3 and T4 levels were measured on day 14 and 42, respectively. **B** Body mass on days 0–42; **C** body mass gain on day 14 and 42 relative to that on day 0; **D** food intake on days 0–42. Data are means ± s.e.m. *, significant difference between the groups (*P* < 0.05), **, *P* < 0.01, ns, non-significant difference (*P* > 0.05)

Forty-eight male striped hamsters, aged 3–4 months, were randomly allocated to one of three groups: 21 °C (21 °C,* n* = 16), 10 °C (10 °C,* n* = 16) or 0 °C group (0 °C,* n* = 16). The hamsters in the 21 °C group were kept at 21 °C for 2 weeks, whereas the hamsters in the 10 °C and 0 °C groups were exposed to 10 °C and 0 °C, respectively, for 2 weeks (hereafter, briefly as cold period). After these 2 weeks, the hamsters in all three groups were exposed to warm temperature for 4 weeks, where the ambient temperature was controlled at 30 ± 1 °C (hereafter, briefly as warm period, Fig. 1A). The pre-experiment performed in the striped hamsters in our lab showed that the animals had consistent increases in energy intake and expenditure, and decreases in fat content after exposure to the cold (10 °C and below) for 2 weeks. Both energy intake and expenditure were consistently decreased in the hamsters exposed to the warm (30 °C) for 3 or 4 weeks or longer than that, which was paralleled with a considerable increase in body fat content. This is the reason why the animals were acclimated to the cold temperatures for 2 weeks and to the warm temperature for 4 weeks.

### Body mass and food intake

Body mass and food intake were measured on a daily basis over the period of the experiment, i.e. 2 weeks of cold + 4 weeks of warm exposure. Food intake was calculated as the amount of food missing from the hopper each day subtracting the orts mixed in bedding.

### Gross energy intake (GEI) and digestibility

GEI and digestibility were measured over the last two days of the cold exposure (day 12–14). The food was provided quantitatively on the hopper on day 12, and 48 h later the food left on the hopper and the food residues mixed with bedding were collected from each cage, during which the feces were also collected. The measurements were repeated over the last two days of the warm exposure (day 40–42). Food residues and feces were dried in an oven at 60 °C for 2 weeks and then were separated manually. The gross energy contents of the food and feces were determined using an oxygen bomb calorimeter (C2000, Germany), strictly according the instructions of the manufacturer. GEI, digestive energy intake (DEI) and digestibility were calculated as follows: GEI (kJ/d) = food intake (g/d) × dry matter content of the diet (%) × energy content of food (kJ/g); Gross energy of feces (GEF) (kJ/d) = dry mass of feces (g/d) × energy content of feces (kJ/g); DEI (kJ/d) = GEI − GEF; Digestibility (%) = DEI/GEI × 100% [43–45].

### RMR and NST

At the end of the cold and warm exposure, RMR was quantified as the rate of oxygen consumption in an open-flow respirometry system (TSE system, Germany). Briefly, air was pumped at a rate of 1000 mL/min (CaloSys-Pump 994620-Air-YP-VC, TSE system) through a cylindrical sealed Perspex chamber (35 cm in length, 8 cm in diameter, and 1748 mL in volume). The air flow rate was controlled by the air control unit (PROCESS CONTROL FOR VALVES, 994620-CFV-08, TSE system), and was measured using a mass-flowmeter (GCM-B5SA-BNOO, 994820-FC-05, TSE system). Gases leaving the chamber were dried using a special drier (TSE system, Germany) and directed through the Oxygen analyzer at a flow rate of 380 mL/min (TSE subsample control unit, TSE system). Oxygen concentration was determined using the Oxygen analyzer (ULTRAMAT/OXYMAT 6, 994620-CS-HSP-01-O_2_CO_2_, Siemens, Germany). Calibration of equipment (CaloSys Calibration Unit) was included in the TSE system, and three gases was used when calibrated (gas 1, N_2_; gas 2, O_2_ 20.95% and CO_2_ 0.05% with N_2_ balance; gas 3, O_2_ 20.00% and CO_2_ 0.95% with N_2_ balance). RMR was measured for 3 h in the chamber at 30 ± 0.5 °C (within the thermal neutral zone of this species), and the measurement of ambient temperature in the chamber was performed using a temperature probe (TSE system). The data were averaged and collected every 10 s by a computer connected analogue–digital converter (TSE system), and the continuous stable minimal recordings over 10 min were taken to calculate RMR [45, 46].

Maximum NST (NST) was determined also using an open-flow respirometry system as the method mentioned above. NST was defined as the maximum rate of oxygen consumption in response to noradrenaline (NE) [8, 47] and was induced by a subcutaneous injection of NE at 25 ± 0.5 °C. The mass-dependent dosage of NE (Shanghai Harvest Pharmaceutical Co, Ltd) was calculated according to the equation: NE (mg/kg) = 6.6 Mb^−0.458^ (g) (Mb, body mass) [48]. NST was calculated from continuous stable maximal recordings over 10 min. All measurements were performed between 10:00 and 16:00. RMR and NST were finally corrected to standard temperature and air pressure (STP) conditions, and were expressed as mLO_2_/h.

### Blood collection and serum T3 and T4 measurements

Animals were sacrificed after the cold exposure (9:00–11:00 on day 14, *n* = 8 for each group), and the warm exposure (9:00–11:00 on day 42, *n* = 8 for each group). The trunk blood was collected, and clotted for 2 h at room temperature, and serum was separated following the centrifugation at 3000 r/min for 15 min, and was stored at − 20 °C. Total T3 and total T4 concentrations were determined in 100 µL serum samples by radioimmunoassay using I^125^ RIA kits (T3, KIP1631, T4, KIP1641, Beijing North Institute of Biological Technology, China), in duplicate determinations following manufacturer's instruction [29]. These assays contained anti-T4 or anti-T3 monoclonal antibodies, respectively (Beijing North Institute of Biological Technology). These kits were validated for the striped hamsters tested by cross activity. Intra- and inter-assay coefficients of variation were < 10% and < 15% for the T3, and < 10% and < 15% for T4, respectively. The functional sensitivity of the T3 and T4 assay was 0.31 nmol/L and 6.44 nmol/L, respectively.

### Body fat content

Interscapular BAT was quickly removed, weighed and immediately frozen using liquid nitrogen after trunk blood was collected. The gastrointestinal tracts (stomach, small and large intestines, and cecum) were separated and removed. Liver, heart, lung, spleen, and kidneys were also removed. The remaining carcass was weighed (to 0.01 g) to determine wet mass and was dried in an over at 60 °C to determine dry mass. Total body fat was extracted from the dried carcass by ether extraction in a Soxhlet apparatus. The percentage of lipid in the initial sample was calculated according to the equation: Fat content (%) = (total fat of carcass/dry carcass mass) × 100% [15].

### Mitochondrial protein content and COX activity

The interscapular BAT was weighed (to 1 mg), and finely dissected, and homogenized (1:15, w/v) with ice-cold isolation buffer containing 250 mM sucrose, 10 mM TES, 1 mM EDTA and 64 µM bovine serum albumin (BSA) (PH 7.2). The homogenate was centrifuged (12,096×*g* for 10 min at 4 °C), and the supernatant was discarded. The pellets were resuspended with an ice-cold medium containing 250 mM sucrose, 10 mM TES, 1 mM EGTA and 64 µM BSA (PH 7.2). After a brief centrifugation (500×*g* for 10 min at 4 °C), the supernatant was transferred into an ice-cold centrifuge tube and was then centrifuged (8740×*g* for 10 min at 4 °C). The pellets (isolated mitochondria) were resuspended in ice-cold suspension buffer (1:1, w/v), containing 100 mM KCl, 20 mM TES and 1 mM EGTA (PH 7.2). Mitochondrial protein concentrations were determined by the Folin phenol method with BSA as the standards [49]. COX activity of tissues was measured with a polarographic method using an oxygen electrode (Hansatech Instruments Ltd., England) [29].

### Real-time RT-qPCR analysis

Total RNA in BAT was isolated using the NRA isolation kit (TAKARA, Japan) and the TissueRuptor (IKA, Germany) for complete sample disruption/homogenization, as described in the manufacturer’s protocol. RNA quantity and purity (OD 260/280 ratio 1.9–2.1) were measured with the spectrophotometer (Puxitongyong-T6, Beijing, China). The cDNA was synthesized from 2 µg of total RNA in a final reaction volume of 50 µL with AMV Reverse Transcriptase (TAKARA) using random primer oligo (dT)_18_. The cDNA sample (2 µL) was taken for the subsequent PCR reaction using gene-specific primers: forward, 5′–GGGACCATCACCACCCTGGCAAAAA–3′, reverse, 5′–GGCTTTCTGTTGTGGCTAT–3′. All PCR reactions were performed in a 20 µL reaction volume (TB Green PrimeScrip RT-PCR Kit, RR086A, TAKARA) on Roche Light Cycler 480 real-time qPCR system (Forrentrasse CH-6343 Rotkreuz, Switzerland), including 10 µL of 2 × SYBR Premix EX Tag TM (TAKARA), 0.4 µL of forward and reverse primer and 2 µL cDNA template. The qRT-PCR measurements for all samples were performed in duplicate and every run included a no-template control. The PCR program started with an initial denaturation at 95 °C for 3 min to activate the Taq polymerase, followed by 40 cycles of 5 s at 95 °C, 30 s at 55 °C and 30 s at 72 °C, followed by thermal denaturation curves, during which fluorescence was measured. A melting curve was constructed to verify the presence of a single gene-specific amplicon and the absence of any primer dimers by heating the samples from 70 to 95 °C in 0.5 °C increments, during which the fluorescence was continuously monitored [50]. All samples were quantified for relative quantity of gene expression by using actin expression as an internal standard: forward, 5′–AAAGACCTCTATGCCAACA–3′, reverse, 5′–ACATCTGCTGGAAGGTGG–3′ [15].

### Statistical analysis

Data were expressed as the mean ± SE and analyzed using SPSS 21.0 statistic software. The Kolmogorov–Smirnov test was used to evaluate the normality of the distribution, and the* F*-test was used to examine the homogeneity of variance, indicating that all data showed a normal distribution and homogeneity of variance, and met the assumptions of ANOVA testing. Effects of temperature on food intake, GEI, DEI, RMR, NST, and body fat, were examined using one-way ANCOVA with body mass as a covariate. Body mass, digestibility, BAT mitochondrial protein content, COX activity, and *ucp*_1_ mRNA expression were examined using one-way ANOVA. The differences in these variates across the groups were analyzed using *S–N–K* post hoc tests following the one-way ANOVA analysis where required. Pearson’s correlation analysis was performed to examine the relationships between fat content and body mass, GEI, RMR, and NST, and BAT COX activity and *ucp*_1_ mRNA expression. We run the ANCOVA with group as the fixed effect and one of tested variable as covariate to examine the effect of group on the correlations. The level of significance was set at* P* < 0.05.

## Results

### Body mass and food intake

Body mass was not significantly different between the three groups prior to the experiment started (day 0, 21 °C, 35.1 ± 1.6 g; 10 °C, 35.3 ± 1.3 g; 0 °C, 34.9 ± 1.3 g; *F*_2,21_ = 0.01, *P* > 0.05), or over the period of cold or warm exposure (day 14, 21 °C, 35.6 ± 1.7 g; 10 °C, 35.5 ± 1.6 g; 0 °C, 35.7 ± 1.3 g; *F*_2,21_ = 0.01, *P* > 0.05; day 42, 21–30 °C, 36.4 ± 1.6 g; 10–30 °C, 37.4 ± 1.7 g; 0–30 °C, 38.5 ± 1.4 g; *F*_2,21_ = 0.47, *P* > 0.05, Fig. 1B). Body mass gain following the cold exposure was not significantly different between 21 °C, 10 °C and 0 °C groups (day 14, *F*_2,21_ = 0.16, *P* > 0.05), while it was considerably increased following the warm exposure, being 3.7 ± 0.7%, 6.1 ± 1.0% and 9.8 ± 1.4% higher, respectively, in 21–30 °C, 10–30 °C and 0–30 °C groups on day 42 relative to day 0 (*F*_2,21_ = 8.43, *P* < 0.01, Fig. 1C).

Food intake was not significantly different between the three groups on day 0 (21 °C, 3.8 ± 0.5 g/d; 10 °C, 3.8 ± 0.3 g/d; 0 °C, 4.1 ± 0.4 g/d). The hamsters consumed considerably more food following the cold exposure, with food intake being higher by 61.8% and 91.8% in the 10 °C and 0 °C groups than that in the 21 °C group on day 14 (21 °C, 3.3 ± 0.2 g/d; 10 °C, 5.4 ± 0.2 g/d; 0 °C, 6.4 ± 0.5 g/d; *F*_2,20_ = 20.33, *P* < 0.01, Fig. 1D). The hamsters consumed considerably less food after exposure to the warm temperature compared to those under the cold temperature. Interestingly, the food intake on day 42 was significantly different among the three groups, and was higher (by 13.6%) in the 10–30 °C and in the 0–30 °C group (by 39.8%) than in the 21–30 °C group (21–30 °C, 3.1 ± 0.1 g/d; 10–30 °C, 3.5 ± 0.1 g/d; 0–30 °C, 4.3 ± 0.2 g/d; *F*_2,20_ = 32.62, *P* < 0.01, Fig. 1D).

### Energy intake and digestibility

**Fig. 2:**
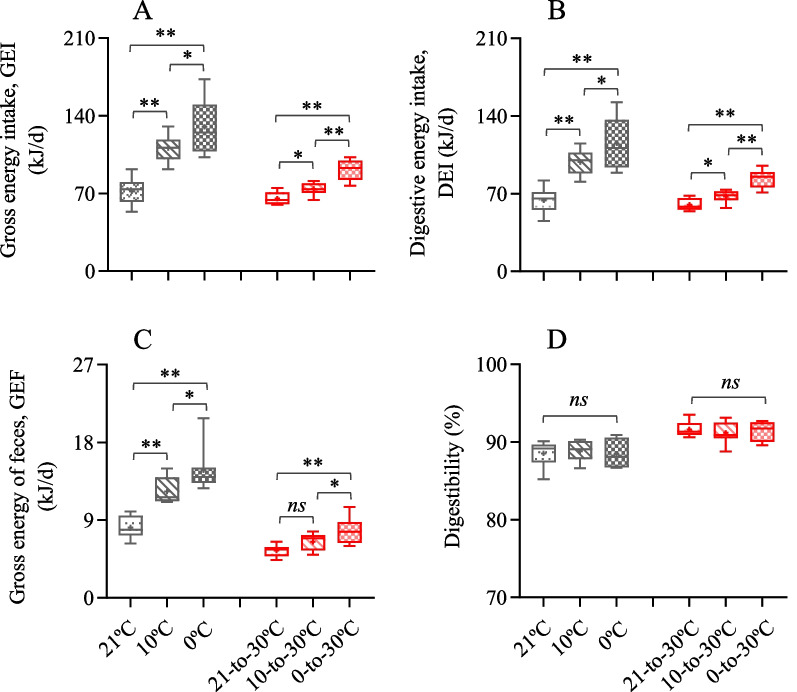
Energy budgets of striped hamsters. **A** Gross energy intake, GEI; **B** digestive energy intake, DEI; **C** gross energy of feces, GEF; **D** digestibility. Animals were exposed to 21, 10 and 0 °C for 14 days and transferred to a warm temperature at 30 °C on days 15–42. Data are means ± s.e.m. *, significant difference between the groups (*P* < 0.05), **, *P* < 0.01, ns, non-significant difference (*P* > 0.05)

GEI and DEI during the cold exposure differed significantly across the three groups, being significantly higher in 10 °C and 0 °C groups compared to 21 °C group (GEI,* F*_2,20_ = 23.11, *P* < 0.01, *S–N–K *post hoc, *P* < 0.01, Fig. 2A; DEI,* F*_2,20_ = 20.93, *P* < 0.01, *S–N-K *post hoc, *P* < 0.01, Fig. 2B). There were considerable reductions in both GEI and DEI following the warm exposure compared to the cold exposure. At the end of warm exposure, GEI and DEI were significantly different across the groups, being higher by 39.2% and 23.1% in the 0–30 °C group compared to 21–30 °C and 10–30 °C groups for GEI, and 38.8% and 23.4% for DEI (GEI,* F*_2,20_ = 32.60, *P* < 0.01, *S–N–K* post hoc, *P* < 0.01; DEI,* F*_2,20_ = 32.16, *P* < 0.01, *S–N–K *post hoc, *P* < 0.01). Consistently, GEF was significantly different across the groups after cold and warm exposure, with the hamsters in 0 °C group producing significantly more feces than the hamsters in 21 °C and 10 °C groups (cold period, *F*_2,20_ = 22.96, *P* < 0.01, warm period, *F*_2,20_ = 7.79, *P* < 0.01, *S–N–K* post hoc, *P* < 0.01, Fig. 2C). Digestibility was not significantly different among the groups either after cold or warm exposure (cold period, *F*_2,21_ = 0.10, *P* > 0.05, warm period,* F*_2,21_ = 0.28, *P* > 0.05, Fig. 2D).

### RMR and NST

**Fig. 3:**
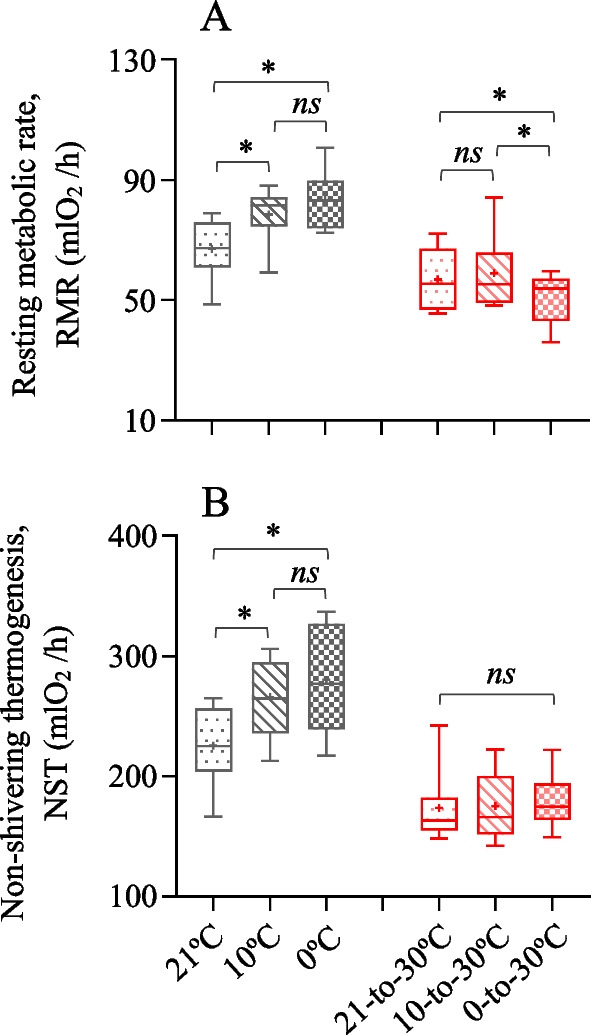
Metabolic thermogenesis of striped hamsters. **A** Resting metabolic rate, RMR and **B** non-shivering thermogenesis, NST. Animals were exposed to 21, 10 and 0 °C for 14 days and transferred to a warm temperature at 30 °C on days 15–42. Data are means ± s.e.m. *, significant difference between the groups (*P* < 0.05), **, *P* < 0.01, ns, non-significant difference (*P* > 0.05)

The RMR during cold exposure was higher by 17.3% and 24.4% in the 10 °C and 0 °C groups than that in the 21 °C group (*F*_2,20_ = 17.28, *P* < 0.01, *S–N–K* post hoc, *P* < 0.05, Fig. 3A). After exposure to the warm temperature, the hamsters in 0–30 °C group showed 10.0% and 13.1% lower RMR than the hamsters in 10–30 °C and 21–30 °C groups (*F*_2,20_ = 8.99, *P* < 0.01, *S–N–K *post hoc, *P* < 0.05). The NST during cold exposure was also significantly higher in the 10 °C and 0 °C groups than that in the 21 °C group (*F*_2,20_ = 15.76, *P* < 0.01, *S–N–K* post hoc, *P* < 0.05), whereas it was not significantly different between the three groups after exposure to the warm temperature (*F*_2,20_ = 0.30, *P* > 0.05, Fig. 3B).

### Body fat

**Fig. 4:**
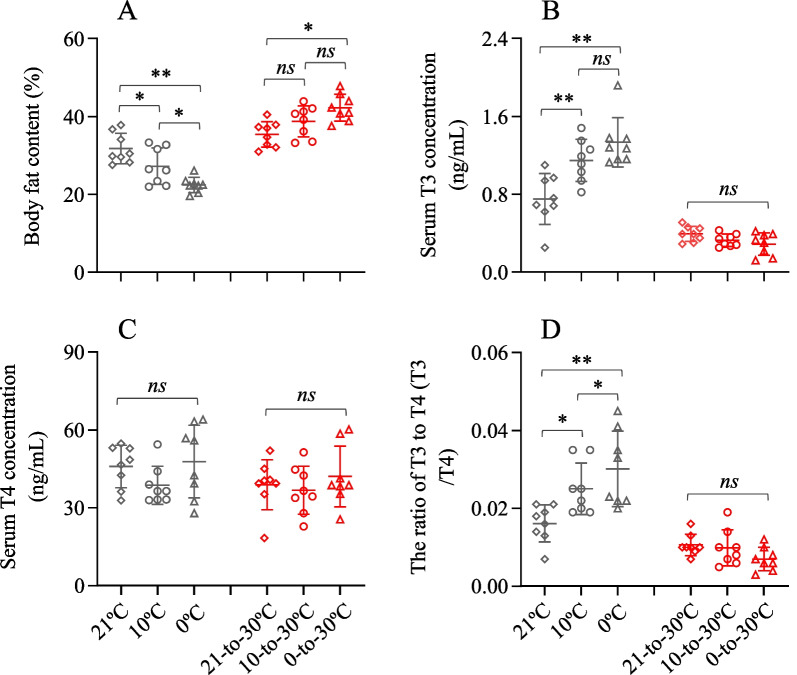
Body fat and thyroid hormones of striped hamsters. **A** Body fat content, and **B** serum T3; **C** serum T4 and **D** the ratio of T3 to T4. Animals were exposed to 21, 10 and 0 °C for 14 days and transferred to a warm temperature at 30 °C on days 15–42. Data are means ± s.e.m. *, significant difference between the groups (*P* < 0.05), **, *P* < 0.01, ns, non-significant difference (*P* > 0.05)

Body fat content of the hamsters during cold exposure was significantly different across the groups, with the hamsters in 0 °C group showing significantly less body fat compared to the 21 °C and 10 °C groups (*F*_2,20_ = 13.95, *P* < 0.01, *S–N–K* post hoc, *P* < 0.01, Fig. 4A). After exposure to the warm temperature, the hamsters considerably increased fat accumulation compared to the hamsters at cold temperatures. The body fat content was not significantly different between the 10–30 °C and 21–30 °C groups, whereas it was significantly higher (by 19.3%) in the 0–30 °C group than that in 21–30 °C group (*F*_2,20_ = 18.84, *P* < 0.01, *S–N–K* post hoc, *P* < 0.05, Fig. 4A).

### Serum T3 and T4

Serum T3 concentration of the hamsters at cold temperatures was higher by 52.7% and 77.5% in the 10 °C and 0 °C groups than that in the 21 °C group (*F*_2,21_ = 11.71, *P* < 0.01, *S–N–K* post hoc, *P* < 0.01, Fig. 4B). After exposure to the warm temperature, no significant difference was observed in serum T3 across the three groups (*F*_2,21_ = 2.89, *P* > 0.05). Serum T4 concentration was not significantly different across the three groups either after cold exposure (*F*_2,21_ = 1.76, *P* > 0.05) or warm exposure (*F*_2,21_ = 0.55, *P* > 0.05, Fig. 4C). The ratio of T3 to T4 during cold exposure was significantly higher in 10 °C and 0 °C groups than that in 21 °C group (*F*_2,21_ = 7.53, *P* < 0.01), whereas the ratio during warm exposure was not significantly different across the groups (*F*_2,21_ = 2.32, *P* > 0.05, Fig. 4D).

### Correlations between body fat content and energy intake

**Fig. 5:**
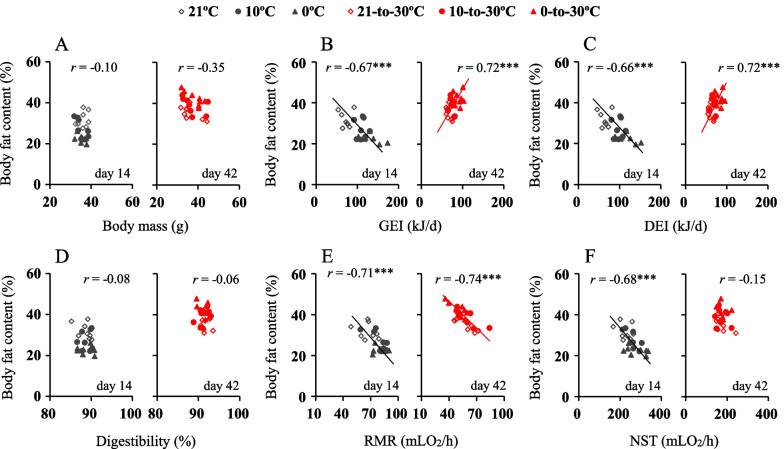
Correlations between body fat content, energy intake and expenditure. **A** Body mass; **B** gross energy intake, GEI; **C** digestive energy intake, DEI; **D** digestibility; **E** resting metabolic rate, RMR, and **F** non-shivering thermogenesis, NST, of striped hamsters. Animals were exposed to 21, 10 and 0 °C for 14 days and transferred to a warm temperature at 30 °C on days 15–42. *, significant coefficients (*P* < 0.05), ***, *P* < 0.001

There were no significant correlations between body fat content and body mass for the hamsters exposed to either cold or warm temperature (Fig. 5A). The body fat content was negatively correlated with GEI and DEI at cold temperatures, with the hamsters showing higher energy intake, but lower fat content, whereas the correlations were not significantly affected by cold temperatures (body fat and GEI, *F*_2,20_ = 2.76, *P* > 0.05; body fat and DEI, *F*_2,20_ = 3.04, *P* > 0.05). After exposure to the warm temperature, there were positive correlations between body fat and GEI, and DEI, by which the hamsters previously acclimated to 0 °C showed higher energy intake and more body fat accumulation than those previously kept at 21 °C, and the correlations were significantly different between the three groups (body fat and GEI, *F*_2,20_ = 4.73, *P* < 0.05, Fig. 5B; body fat and DEI, *F*_2,20_ = 5.02, *P* < 0.05, Fig. 5C). The correlations between body fat content and digestibility were not statistically significant either at cold or warm temperature (Fig. 5D).

### Correlations between body fat content and energy expenditure

The body fat content was negatively correlated with RMR at the cold and warm temperatures, with the hamster characterized by higher RMR showing lower body fat content (Fig. 5E). The body fat content was also negatively correlated with NST at cold temperature, whereas the correlation was not statistically significant at warm temperature (Fig. 5F). The correlations between body fat and RMR, and NST, were significantly different between the groups at the cold (body fat and RMR, *F*_2,20_ = 5.09, *P* < 0.05; Body fat and NST, *F*_2,20_ = 6.46, *P* < 0.01), and at the warm temperature (body fat and RMR, *F*_2,20_ = 12.75, *P* < 0.01; Body fat and NST, *F*_2,20_ = 8.62, *P* < 0.01).

### BAT COX activity and ucp_1_ expression

**Fig. 6:**
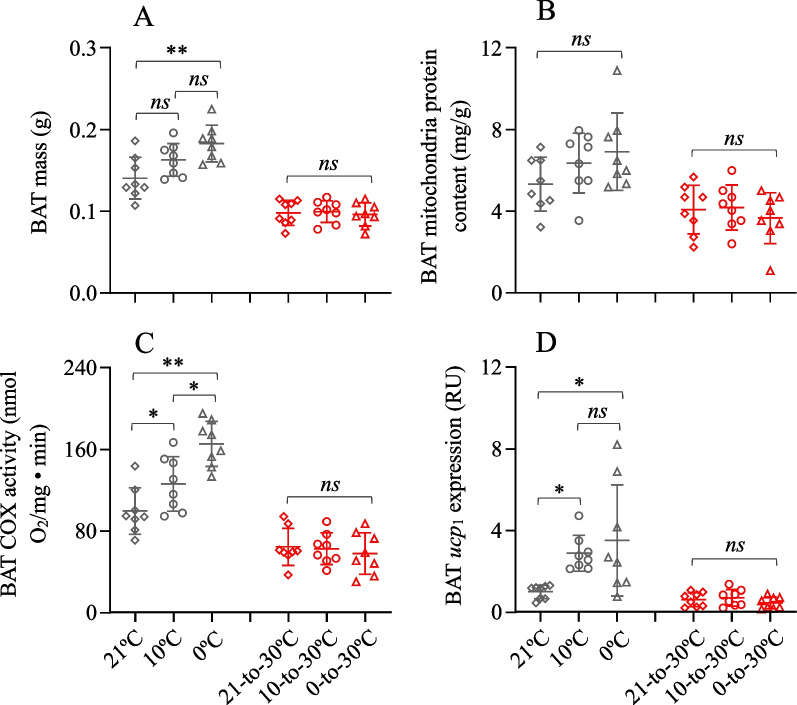
The thermogenic activity of brown adipose tissue (BAT) of striped hamsters. **A** BAT mass; **B** mitochondria protein content; **C** cytochrome c oxidase activity, COX, and **D** the *ucp*_1_ gene expression. Animals were exposed to 21, 10 and 0 °C for 14 days and transferred to a warm temperature at 30 °C on days 15–42. Data are means ± s.e.m. *, significant difference between the groups (*P* < 0.05), **, *P* < 0.01, ns, non-significant difference (*P* > 0.05)

The BAT mass of cold exposed hamsters was significantly different between the 21 °C and 0 °C groups, being higher by 30.2% at 0 °C compared to that at 21 °C (*F*_2,21_ = 6.92, *P* < 0.01, *S–N–K* post hoc, *P* < 0.01, Fig. 6A). After exposure to the warm temperature, BAT mass did not significantly differ across the three groups (*F*_2,21_ = 0.12, *P* > 0.05). BAT mitochondria protein content was not significantly different across the three group either after the cold or warm exposure (cold, *F*_2,21_ = 2.10, *P* > 0.05; warm, *F*_2,21_ = 0.42, *P* > 0.05, Fig. 6B). BAT cox activity was significantly increased following the cold exposure, being higher by 26.7% and 66.1% in the 10 °C and 0 °C groups, respectively, than in the 21 °C group (*F*_2,21_ = 6.92, *P* < 0.01, *S–N–K* post hoc, *P* < 0.01, Fig. 6C). There was no group difference in BAT cox activity after exposure to the warm temperature (*F*_2,21_ = 0.29, *P* > 0.05). Similarly, BAT *ucp*_1_ gene expression was significantly increased following the cold exposure, being higher by 188.4% and 250.8% in the 10 °C and 0 °C groups than in the 21 °C group (*F*_2,21_ = 4.98, *P* < 0.05, *S–N–K* post hoc, *P* < 0.05, Fig. 6D), whereas it was not significantly different between the three groups following the warm exposure (*F*_2,21_ = 1.02, *P* > 0.05).

**Fig. 7:**
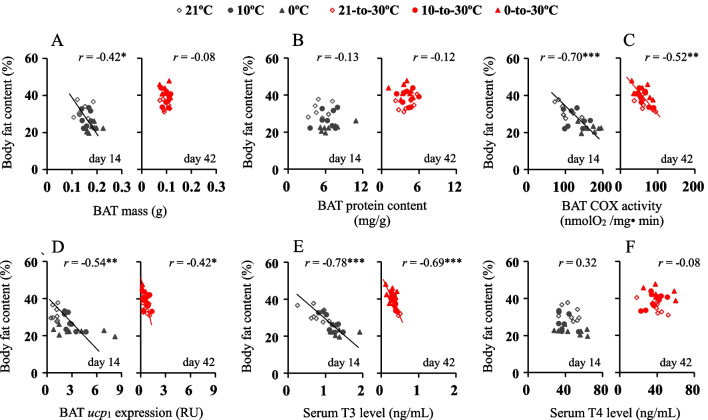
Correlations between body fat content and BAT thermogenic activity. **A** BAT mass; **B** mitochondria protein content; **C** cytochrome c oxidase activity, COX; **D** gene expression of uncoupling protein 1, *ucp*_1_, **E** serum T3 levels, and **F** serum T4 levels. Animals were exposed to 21, 10 and 0 °C for 14 days and transferred to a warm temperature at 30 °C on days 15–42. *, significant coefficients (*P* < 0.05), ***, *P* < 0.001

Body fat content was negatively correlated with BAT mass at cold, with the correlations being significantly affected by the groups (*F*_2,20_ = 8.73, *P* < 0.01), whereas the correlation was not statistically significant at warm (Fig. 7A). There were no significant correlations between body fat and BAT mitochondria protein content under either cold or warm conditions (Fig. 7B). Body fat content was negatively correlated with BAT COX activity, and *ucp*_1_ gene expression at the cold and warm (Fig. 7C, D). The correlations between body fat and COX were not significantly affected by groups at the cold (*F*_2,20_ = 2.30, *P* > 0.05), but they were significantly different between the groups at the warm (*F*_2,20_ = 8.41, *P* < 0.01). The correlations between body fat and *ucp*_1_ were significantly different between the groups at the cold (*F*_2,20_ = 6.56, *P* < 0.01), and at the warm (*F*_2,20_ = 6.63, *P* < 0.01). There were negative correlations between body fat and serum T3 at both temperatures, whereas the correlations were not significantly different between the groups either at the cold (*F*_2,20_ = 2.55, *P* > 0.05) or at the warm temperature (*F*_2,20_ = 3.39, *P* > 0.05, Fig. 7E). The correlations between body fat content and serum T4 levels were not statistically significant under either cold or warm condition (Fig. 7F).

## Discussion

In the present study, the striped hamsters that were previously exposed to 0 °C had significantly more fat accumulation after transition to the warm temperature compared to those hamsters previously exposed to 10 °C or 21 °C. Consistently, desert hamsters (*Phodopus roborovskii*) [51], Brandt’s voles [52], prairie voles (*Microtus ochrogaster*) [53] and Mongolian gerbils [20] decrease their body fat in response to cold temperatures. Therefore, it is possible to hypothesize that in some rodents there is a general response to cold temperature on a basis of body fat regulation, and vice versa. However, the effect of ambient temperature should be distinguished from the seasonal effect on the body fat. For example, some non-hibernating rodents living in temperate zones have annual body fat cycles, showing naturally occurring decreases in adiposity in the fall or winter, e.g. Siberian hamsters (*Phodopus sungorus*) [54], Djungarian hamsters [55], Brandt’s voles [19], meadow voles (*Microtus pennsylvanicus*) [56], Maximowiczi's voles (*Microtus maximowiczii*) [57] and Mongolian gerbils [20]. The seasonal effects reflect the mixed factors in the wild rather than the ambient temperature only. In addition, a seasonal fattening is observed in a variety of small mammals showing hibernation (e.g. edible dormice (*Glis glis*) [58], ground squirrels (*Citellus townsendi*) [59] and Daurian ground squirrels (*Spermophilus dauricus*) [60]), which suggests that the fattening is also induced by the mixed factors, possibly including the ambient temperature. Therefore, the temperature effect on the body fat appears species specific rather than consistent across all animal species. In this study, the considerable fat accumulation in the striped hamsters under the warm condition may reflect the temperature of prior cold acclimation.

The variations in body fat are the result of the trade-off between energy intake and expenditure. Hyperphagia is one of the most likely factors inducing an imbalance, resulting in excess energy intake and consequently increased fat accumulation [61]. In contrast, in the present study the striped hamsters at the cold temperatures, i.e. 0 °C and 10 °C, consumed significantly more food, whereas they showed less fat content, than those kept at 21 °C. Small mammals usually increase food intake under cold conditions, while consuming less food after being acclimated to warm conditions [2, 14, 62–64]. The data from this study not only show a general pattern regarding the food intake in response to warm temperature, but also indicate that there may be no positive link between energy intake and body fat content in the hamsters transferred from the cold to warm.

**Fig. 8:**
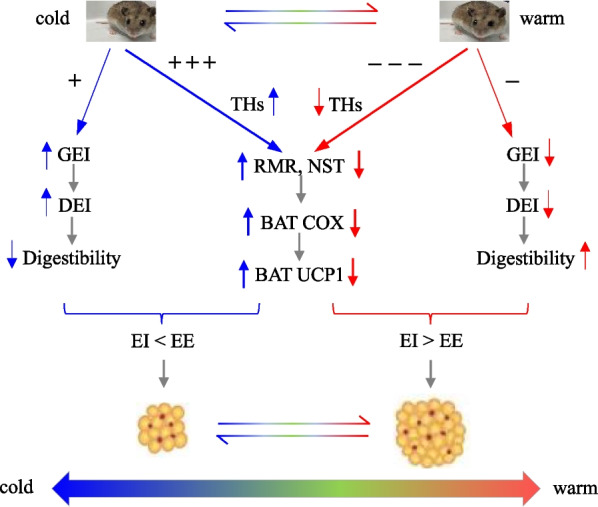
The transition from cold to warm increases fat accumulation. Animals at cold considerably increase metabolic thermogenesis far more than the increases in energy intake, resulted in fat mobilization. After being transferred from cold to warm, animals considerably decrease energy intake and thermogenesis too. However, the decreased metabolic thermogenesis could not compensate for the decreased energy intake, consequently resulted in positive energy balance and fat accumulation. + Indicates positive effect of cold exposure; − indicates negative effect of warm exposure. Blue arrow up, increase; and red arrow down, decrease. THs, thyroid hormones; GEI, gross energy intake; DEI, digestive energy intake; RMR, resting metabolic rate; NST, nonshivering thermogenesis; BAT, brown adipose tissue; COX, cytochrome c oxidase; UCP1, uncoupling protein 1; EI, energy intake; EE, energy expenditure

Consistent with energy intake, in this study the energy spent on metabolic thermogenesis, indicated by RMR and NST, was significantly higher in the cold-exposed hamsters than those at the warm temperature. RMR and NST are significantly up-regulated in cold-exposed animals to compensate heat loss, and consequently to maintain normal thermoregulation [10, 62]. In contrast, in the animals within their thermal neutral zone, the metabolic thermogenesis in the BAT decreases to the minimum as it is not important and necessary, which is consistent with that observed in the striped hamsters under the warm condition in this study. Further, we observed negative correlations between body fat content and RMR, and NST. These findings suggest that the positive energy balance, resulting from the decreased metabolic thermogenesis rather than energy intake, contributes to the increased body fat content in the hamster transferred from cold to warm condition (Fig. 8).

BAT has been proposed to prevent body fat accumulation by burning off excess energy to maintain energy balance, and thus also plays an important role in body mass and body fat regulation [33, 34]. In our study, BAT mitochondria protein content, COX activity and *ucp*_1_ expression were considerably decreased in the hamsters transferred to the warm temperature compared to that in the hamsters that were previously exposed to cold temperatures. Since the NST in BAT is not used any more under the warm conditions (i.e. the temperatures within the TNZ), the activity of thermogenic metabolism in BAT is significantly decreased compared to that under the cold conditions. These findings are consistent with the studies that are previously performed in other animals, such as root voles (*Microtus oeconomus*) [62], Djungarian hamsters [1, 2] and Mongolian gerbils [65]. However, it is important to note that it is not only BAT thermogenesis but also a combination of NST via different mechanisms and shivering is used by different small mammal species to cope with ambient temperature [14, 26]. In our study, BAT COX activity and UCP1 expression were negatively correlated with body fat content, suggesting a possible link between BAT activity and fat accumulation. Consistently, BAT UCP1 expression was significantly down-regulated in Brandt’s voles after being transferred from cold (5 °C) to warm condition (23 °C), which was in parallel with the increases in body mass and body fat mass [52]. It has been also reported that increasing the daily hours of light exposure increases body fat through attenuation of BAT activity [66]. This finding suggests that the changes in body fat in response to variations of environment factors, such as temperature and photoperiod, are likely associated with BAT activity. Further, BAT activity indicated by the COX activity and UCP1 expression may be involved in the increased body fat accumulation (Fig. 8).

We observed that serum T3 levels were significantly increased in the hamsters exposed to 10 °C and 0 °C compared to that at 21 °C, and was considerably decreased after the transition to the warm temperature. Consistently, the changes in THs levels in response to either cold or warm temperature are observed in other animal species, such as tree shrew (*Tupaia belangeri*), greater vole (*Eothenomys miletus*), Brandt's vole, Mongolian gerbil, Syrian hamster (*Mesocricetus auratus*), Daurian ground squirrel (*Spermophilus dauricus*) and plateau pika (*Ochotona curzoniae*) [67–69]. Interestingly, in this study we observed that serum T3 levels were changed in a way in contrast to body fat content in the hamsters either exposed to cold or transferred to warm temperature. There were strong negative correlations between serum T3 levels and body fat content. Similarly, serum T3 levels are considerably decreased in Swiss mice following a gradient rise in ambient temperatures from 5 to 35 °C, which is paralleled with a significant reduction of BAT COX activity, but a considerable increase in body fat deposit [70]. These findings suggest that the reduction of THs in stimulating metabolic thermogenesis, including RMR and the NST in BAT but not excluding the NST in skeletal muscle, may be associated with fat accumulation (Fig. 8). The ecological mechanism which favors the increased fat accumulation in warm ambient temperatures after cold exposure would be of interest and significance, however it is unfortunately not examined in the present study.

## Conclusion

In summary, the striped hamsters showed a significant reduction in energy intake, but had considerable accumulation of body fat under the warm conditions compared to those under the cold conditions. Further, body fat content was negatively correlated with BAT COX activity and *ucp*_1_ expression, and serum T3 levels. These findings suggest that the positive energy balance under the warm condition resulting from the decreased metabolic thermogenesis contributes the fat accumulation (Fig. 8). The hamsters that were previously exposed to the cold condition had more fat accumulation after being transferred to warm condition, suggesting that the fat accumulation under warm conditions is determined by the extent of previous cold exposure.

## Data Availability

All data used in the analyses are provided in the manuscript, or provided under request.
